# A Monster of the Sea

**Published:** 1881-10

**Authors:** 


### A MONSTER OF THE SEA.

A Gloucester (Mass.) fisherman said to a New York reporter, while examining a squid at a Rockaway museum:

"I've been round the world, seen sharks, whales and big snakes, but a big squid when he's cornered is about the worst-looking creature you want to see. Generally their body is about ten feet long, looks like a grayish-white bag, with a tail like a big arrow-head. The head is small, but the eyes are about as big as a large saucer or plate, and black and staring. When you catch a glimpse of them eyeing you out from among their arms, I tell you it makes a man wish he hadn't come. The arms, ten of them, branch from the head, eight short ones about fifteen feet, and two long ones from thirty to forty, depending, of course, upon the size of the squid. Eight of them are lined with suckers, each one ranging in size from a ten-cent piece up to a half-dollar. They are like so many air pumps. In each one is a ring of bone with edges like a saw. These are pressed into you, and the air is sucked out, which, of course, forces the teeth of the saw in, and you can imagine the effect of hundreds of these flying around and striking on all sides. The long arms only have their suckers confined to the ends, which are flattened out. Between all these arms is the mouth, which has two beaks just like a parrot's, only larger, and the upper one sets into the under so they can nip a piece out of an oar blade as easy as to say the word.

"Do they swim? Yes, and backward, too, dragging the arms after them and going like lightning. Sometimes they jump right out of water and come down as slick as a flying fish. The first one I ever tackled was just above Trinity Bay, Newfoundland. We saw something in near shore, and a couple of us jumped into a dory and pulled over to it. When we got near, a big wave tossed us right on top of it, and the first thing I knew I got a shot of water and ink (you know they spurt ink from an ink bag) fair in the face, and by the time I wiped it off the squid was half aboard us. It flung five of its arms over, and one struck my mate on his bare arm and nearly hauled him over. I grabbed the ax and managed to cut two of the arms, when another got around my leg and hauled me off my feet; down I went into the boat, and I believe that's the only thing that saved us, as my hand landed on a big boat hook. I lay on my back, the boat half full of water, and jammed that hook right through the ugly creature's eyes, and, as my mate had put an oar through it, it slipped into the water. All this time, mind you, it was fuming and spurting water and ink; but it was only about half a fathom of water, and I stuck the boat hook in it again. After we had bailed out the boat we made the squid fast by the painter, towed it aboard and cut it up for bait, after we had measured it. From the tip of the long arms to the end of the tail the line gave fifty-one and a half feet. We packed it in a tub that was made to hold exactly 900 pounds of cod, and it filled it. I wouldn't tackle one again like it for the proceeds of a whole season.

"Why, everywhere a sucker had struck my mate's arm it looked as though a red-hot iron had been pressed on and sunk in, and where they had been torn away the flesh had gone, too. He was laid up a month. I had a heavy pair of boots on, and the leather showed the marks, as if they had been cut with a penknife.

"Yes (in answer to a question), most all the Gloucester men can tell big stories about squids. Captain Collins, now one of the United States fish commissioners, used to run the schooner Howard, and they captured five in one day, averaging from thirty-five to forty-five feet on an estimate, and weighing about a thousand pounds a piece. Some difference between them and this monster, that we are money out on."

This account was not exaggerated, as any one may prove by paying a visit to the zoological museum of Yale College, where Professor Verrill has the finest collection of these creatures in this or any country. A few years ago they were not believed in, and the strange tales of Hugo were the only hints of their existence; but one was washed ashore on the Newfoundland coast, and fortunately fell into the hands of the Rev. Dr. Harvey, who sent part of it to the Smithsonian Institute, and thus their existence became assured and credited by many who some years back classed them with the sea-serpent.

At certain seasons they are more frequent than at others, and as they are only found or seen when mutilated, living at other times in the deep sea, it is supposed that they become injured in the breeding season; or, perhaps, at certain times parasitic animals are more frequent. 1875 was a season extremely notable in this respect, and numbers were seen floating on the surface, food for birds, or partly dead and mutilated. Others were found along the coast washed among the breakers, where they swung, hanging by their two long tentacles, which were fastened to the rocks, answering the purpose of cables to the living ship on a lee shore.

A famous place for them seems to be the Flemish Cap, a bank to the northeast of the Grand Banks. Portions of these monsters have been found in whales, that indicated animals nearly one hundred feet long and twenty-five hundred pounds in weight. These animals are not new to the geologists. Their fossil beaks and ink bags are frequently found in the strata of the recent formations, the ink being so well preserved that it was formerly used as the sepia of commerce, and a writer has penned a history of living squids with the ink of one that perished tens of thousands of years in the past. Earlier forms of the squid appeared in shells, and these fossil coverings are frequently found almost as large as a cart wheel, while some of the straight-shelled varieties reached a length of fifteen feet, and, according to some authorities, thirty feet. Imagine a shell thirty feet in length propelled by a battering ram through the water, waving its snake-like arms; a fitting forefather of the giant squid of to-day, the architenthis of the scientific world.

---

The Grease Tree of China.—Mention has been made in these columns of the value of the grease tree of China as a lubricant. It is said that large forests of this vegetable lubricant are to be found there, and they form the source of a considerable local traffic. This tree not very long ago was imported into India, and it is said that the experiment of cultivating it there has proved quite successful. In the Punjaub and northwestern provinces generally, it grows as rapidly and vigorously as in the native soil, and there are already thousands of trees on the government plantations yielding tons of seeds, admirably adapted to a variety of commercial purposes. Dr. Jameson, a chemist in the Punjaub, has prepared a quantity of grease from this tree, and has forwarded on trial a portion of it to the Punjaub railway to have its qualities tested in a practical manner as lubricating matter for those parts of the machinery constantly exposed to friction. The grease thus obtained forms an excellent tallow, burning with a clear, brilliant and, what is infinitely more to the purpose, a white light, and at the same time emitting not a trace of any unpleasant odor or of the ordinary disagreeable accompaniments of combustion.—*Pacific Rural Press.*

---

Again we are compelled to intimate to mountain resort correspondents that snakes under twelve feet in length can get no puffs from this paper. A word to the wise.—*Boston Globe.*

